# Barriers of Ukrainian refugees and migrants in accessing German healthcare

**DOI:** 10.1186/s12913-024-11592-x

**Published:** 2024-09-24

**Authors:** Karina Davitian, Peter Noack, Katharina Eckstein, Jutta Hübner, Emadaldin Ahmadi

**Affiliations:** 1 Klink für Innere Medizin II, Universitätsklinikum Jena, Am Klinikum 1, 07747 Jena, Germany; 2 Lehrstuhl für pädagogische Psychologie, Humboldtstr. 27, 07743 Jena, Germany

**Keywords:** Ukraine, Refugees, Migrants, Barriers, Healthcare system, Accessibility, Qualitative research, Germany

## Abstract

**Background:**

This study focused on Ukrainian refugees and migrants, a population that, with an ongoing war, is expected to grow in Germany. Over 1 million Ukrainians with exceptional legal status and access to public insurance in Germany significantly burden governmental services, especially German healthcare. It is thus essential to facilitate their integration into the healthcare system and ensure its proper usage. Identifying the obstacles Ukrainian refugees and migrants encounter while accessing healthcare services is crucial to ease their integration.

**Methods:**

A qualitative study was conducted from February 2023 to April 2023. Thirty semi-structured interviews were performed with Ukrainian migrants and refugees. The interviews were transcribed verbatim, organized, and categorized. Thematic analysis was performed to identify barriers related to the use of German healthcare services. To assess possible differences in the experiences of Ukrainian refugees and migrants, the responses of these two groups for each topic were analysed separately.

**Results:**

Ukrainian migrants and refugees experience similar barriers while accessing German healthcare services. Predominantly, language barriers and a lack of understanding of the German healthcare system posed the main barriers in both groups. Additionally, structural challenges, such as differences in referral processes, appointment scheduling, and consultation duration, presented further challenges.

**Conclusion:**

This research study emphasizes the importance of addressing cultural and structural barriers to improve healthcare accessibility and utilization for Ukrainian refugees and migrants in Germany to better facilitate their integration into the healthcare system.

**Supplementary Information:**

The online version contains supplementary material available at 10.1186/s12913-024-11592-x.

## Background

With the Russian offensive war against Ukraine, Europe experienced one of the most significant movements of refugees since the Second World War. UNHCR estimates that as of July 2024, more than 6.5 million Ukrainians have fled since 24 February 2024. Many refugees have found shelter in neighbouring countries, mainly in Poland (more than 5.4 million people), Slovakia, Romania, and Moldova [1]. Germany has taken in a considerable number of those seeking protection, with 1,045,185 refugees from Ukraine recorded in the Central Register of Foreigners by the end of 2022 [2]. As martial law prohibits men between 18 and 60 from leaving Ukraine, most refugees are women, children, and the elderly.

Many of these people require medical care upon arrival and throughout the rest of their stay. Especially elderly and people with disabilities depend on continuous medical care [3]. With the activation of Directive 2001/55/EG on temporary protection, the legal status of Ukrainian refugees in Germany changed, so they do not have to go through the asylum procedure and can directly access social benefits and state insurance [4]. However, formal entitlement to the healthcare system often does not solve all healthcare problems.

Existing literature shows that refugees, asylum seekers, and migrants face significant challenges in accessing healthcare in host countries. Key barriers include legislative and administrative obstacles, language barriers, lack of cultural mediation, limited knowledge of the health care system, and breaks in the continuity of care [5–7]. Despite legal protections, access to healthcare varies across countries, influenced by national policies [8, 9].

Recent studies assessed perceived barriers and healthcare needs among Ukrainian refugees in Romania. Common barriers encountered included language barriers and a general lack of understanding of the healthcare system. Especially vulnerable groups such as elderly refugees, people with disabilities, and pregnant women faced considerable obstacles in accessing health services [10]. Structural barriers reported were issues with insurance coverage, difficulties locating a specialised doctor, bureaucratic hurdles, long waiting lists, and a lack of trust in physicians [11].

A 2022 quantitative study investigated the challenges faced by general practitioners (GPs) in Germany when interacting with Ukrainian refugees. GPs identified several common challenges, including communication barriers due to a lack of language skills or lack of access to professional interpreters, missing information on refugees’ previous medical conditions, and unrealistic expectations of healthcare services such as routine unsubstantiated blood tests, thyroid tests, and multivitamin prescriptions [12].

Health care is fundamental to the successful integration of immigrants. People who are impaired or burdened by health problems are hindered in their integration. These can be their own conditions as well as illnesses of relatives or individuals from their close social environment. The illness promotes marginalization, and marginalization promotes the illness, creating a “vicious circle. " On the other hand, successful integration is essential for adequate healthcare, which is often compromised by insufficient access. Access to sufficient health care should be considered equally important as housing and education for the well-being and, thus, integration of migrants [13].

It is important to note that ‘access’ is a broad and ambiguous term. One component of this term concerns whether the person has the legal right to medical help. The other component concerns whether the person seeking help can interact with the health system. “Access” depends on many factors, some related to the health system, others to the person concerned. Factors that can complicate access include insufficient understanding of the healthcare system and lack of knowledge on how to claim healthcare services [13]. Studies of Polish labour migration to the United Kingdom and Norway show that migrants often do not know what medical services they are entitled to in the country of settlement [14]. According to Penchansky & Thomas, access to health care should be a “fit” between the patient and the health care system [15] whereby the patient identifies their health problems, searches for appropriate health care services, uses them and fulfils his medical needs.

On the other hand, health services should be accessible, suitable, available, and adequate [16]. Governments frequently depend on preexisting structures and communication channels, failing to adapt to the needs of refugee populations. Thus, formal entitlement to health services does not ensure that refugees have full access to existing services [17, 18].

Among a growing body of literature on immigrants’ experiences with healthcare in host countries, little is known about Ukrainian refugees’ and migrants’ experiences with the German healthcare system. Since the beginning of the war, the number of Ukrainian citizens in Germany has increased dramatically. According to data from the Federal Statistical Office, approximately one Million Ukrainians were living in Germany as of 30 September 2022, almost seven times more than at the end of February 2022 [19]. Previous reports suggest that Ukrainian refugees have a low intention to permanently settle abroad, as they are hopeful of returning to their home country [20]. This may impact their integration motivations. However, a 2024 intentions survey indicates a growing uncertainty due to the ongoing war, as the proportion of refugees planning or hoping to return to Ukraine in the future has slightly decreased compared to one year ago [21] The unforeseeable end of the war suggests that integration is likely to become more relevant for Ukrainian refugees.

The aim of this article is to assess the challenges and barriers faced by Ukrainian refugees and migrants in accessing healthcare in Germany to identify potential means to improve policies and programs.

## Methods

To assess the experience of Ukrainian migrants and refugees with the German healthcare system, we conducted 30 semi-structured interviews with members of the Ukrainian diaspora. The category “migrants” refers to individuals who had a residence permit and relocated to Germany before the outbreak of the war. This group includes permanent migrants, family reunification migrants, students, and labor migrants. The “refugee” group includes all participants under the temporary protection Directive. We analyzed the indicators for understanding the varied experiences and challenges faced by the two groups, allowing for a comprehensive comparison beyond healthcare access alone based on relevant literature including socioeconomic status [22], housing situation [23, 24], social integration and support networks [25], access to employment opportunities [26]and cultural adaptation and identity [27]. The analysis showed no significant differences between the two groups across these domains, suggesting similar levels of integration and adaptation challenges in the German context.

During the interview process, data collection and analysis alternated. After each interview, the first author transcribed it verbatim, noting important themes before conducting subsequent interviews. Based on identifying novel topics, adjustments to the interview guide were made. The recruitment and interview process was concluded when subsequent interviews no longer introduced new themes or insights, indicating sufficient data saturation.

The English version of the interview guide is available in the supplementary files; see Additional file 1.

### Participants

The study was performed in Germany in 2023. Most participants were recruited online and volunteered in response to study listings posted throughout social media groups targeted at Ukrainian refugees and migrants. Another three participants self-selected in response to postings provided through NGOs, and three participants were recruited through snowball sampling. At the time of the interviews, most participants were residing in Berlin, while others lived in Hamburg and the federal states of Brandenburg, Saxony-Anhalt, Saxony, Thuringia, and North Rhine-Westphalia. Participation was voluntary; written informed consent to participate was obtained from all individual participants involved in the study. Before the interview, participants could choose to be interviewed in either Ukrainian or Russian language. All interviews were conducted by the first author, who is a native Ukrainian and Russian speaker. The interviews were primarily conducted online using the Platform Google Meets and telephone calls, and two interviews were performed in person.

### Data analysis

Two out of 30 interviews were excluded from the final analysis due to the interviewee’s mental conditions, as the provided answers were unreliable for inclusion in the data set. The interviews lasted 20 to 60 min. All conversations were digitally recorded and transcribed verbatim. The data was organized using Microsoft Excel. The prior thematic categorization helped identify the core topics and classify the issues mentioned. During the data analysis, the four-eyes principle was applied to facilitate the reliability and accuracy of the research findings.

## Results

Four main themes emerged from our research: understanding the German healthcare system, barriers to accessing German healthcare, previous experience with the healthcare system and suggested improvements. Our findings demonstrate that Ukrainian migrants and refugees face similar obstacles when accessing German healthcare services; therefore, the results for these two groups are presented together. The demographics are presented in Table 1.

**Table 1:** Sociodemographic characteristics of study participants

Characteristic	*n*	%
Sex		
Female	21	75
Male	7	25
Living situation		
Alone	7	25
With family	21	75
Age		
20-30	11	39
31-40	10	36
>40	7	25
Length of stay in Germany		
½-1 years	11	40
2-5 years	8	30
>5 years	8	30
Status		
Refugee	11	39
Migrant	17	61
Highest educational level		
Vocational school	3	11
Bachelor degree	11	39
Master degree	14	50
Spoken Languages		
Ukrainian	28	100
Russian	25	89
English	22	79
Intermediate	5	
Advanced	17	77
German	25	89
Beginner	10	40
Intermediate	4	16
Advanced	11	44
Occupation		
Unemployed	9	32
Student	3	11
Employed	15	54
No answer	1	4
Religion		
Atheist	19	68
Christian orthodox	5	18
Ukrainian Greek	4	14
Catholic Church		

The study findings show that language barriers and a lack of understanding of the German healthcare system posed the main obstacles. Additionally, adapting to the new healthcare system and structural challenges, such as differences in referral processes, appointment scheduling, and consultation duration, presented further challenges. We found that bureaucratic challenges in obtaining health insurance presented a unique challenge for the refugee group, especially among vulnerable individuals. Further elaboration of these findings will be provided below.

### Obtaining information on health topics and understanding the German healthcare system

Participants were asked about their sources of health-related information and how they accessed it. Only a few respondents received medical information directly from their German healthcare providers. Respondents with limited knowledge of the German language used various strategies to obtain information and communicate with medical practitioners. Many participants consulted Russian-speaking specialists in Germany or contacted their general practitioners in Ukraine. One female refugee participant in her late 40s described her experience:

In the first place, if I have questions about my health, I call my Ukrainian GP. Considering my health issues, I can also talk to my friends who have been living in Germany for a long time, for about 20 years […] I don’t trust local doctors that much.

Several respondents sought information from Ukrainian friends or family members who spoke German and used their assistance as interpreters during consultations. The participants widely used the internet as a source of medical information and engaged with online Ukrainian communities on Facebook and other social media platforms for resources related to health, illness, and treatment. During the interview, a woman in her early thirties shared her challenges navigating the German healthcare system.

I am not aware of how the German medical system works; most of the information I get through internet forums or my own experience. Written blogs, YouTube blogs […] I started reading English-language articles, but they are irrelevant and do not describe what I need.

The lack of understanding of the German healthcare system was frequently cited as a significant challenge among the participants. A female participant in her late 40s highlighted this issue:

I don’t know how the system works; I feel insecure in any situation related to medicine.

Another female participant in her twenties described the challenges she encountered, particularly during the initial six months of her residency in Germany:

Very often, I found that I had unintentionally interrupted some processes because I was unaware they existed. For instance, I would arrive somewhere and be asked for a referral from my family doctor. And I didn’t even know that a referral was needed.

This statement emphasizes the importance of understanding how the healthcare system operates. It highlights the potential adverse effects of lacking knowledge about the system, such as ineffective engagement with healthcare services, which could result in failing to obtain necessary medical care. Many participants noted the necessity for more accessible information about the healthcare system. A woman in her thirties elaborated:

I realised that the German medical system is difficult for me because I don’t understand how it works. And I would still like to have some systemic information before the visit to understand where to go […] What I lack is an outline of what to do before going to the doctor […] it is organisational issues that cause me the most stress.

The lack of understanding of how health insurance works created additional barriers for some participants. It’s important to note that, unlike in Germany, there is no mandatory health insurance in Ukraine, and private health insurance, while available, is not widespread. A female participant in her mid-20s shared her experience:

Another barrier for me is that I don’t understand where, when and what my insurance covers, what tests or examinations are covered.

Participants often mentioned the difference between the healthcare systems in Germany and Ukraine. Several participants conveyed that the Ukrainian healthcare system concentrates more on providing preventive care and is usually perceived by Ukrainians as a service sector. Appointments are available on short notice, physicians spend at least an hour for a single appointment, and preventive lab testing and check-ups are accessible from an early age.

Trust was also a recurring theme among the participants, discussed in various contexts. Many participants expressed having trust in their physicians, although some specified that they trusted their doctors in Ukraine more than those in Germany. Several participants reported overall low levels of trust in medical professionals and expressed that they considered themselves to be their own best advisors. Due to previous negative experiences, some participants revealed trust issues and a lack of confidence in the German healthcare system. A Ukrainian student in her mid-20s explained that it was difficult to establish trust with the German doctors due to the short duration of the consultation:

What annoys me most is the minimal duration of consultations with the doctor. They are very brief, lacking thoroughness and interpersonal comfort […] When you visit a doctor, you want to feel trust, but here, it is difficult to build trust.

For some individuals, past negative experiences have led them to avoid the German healthcare and seek medical assistance in Ukraine. During an interview, a female participant in her late 20s, who had been residing in Germany for five years, shared her perspective.

Can I be honest with you? I’m not satisfied with the health care here, but I wouldn’t say that I often seek medical services. I usually try to either treat myself or go to Ukraine.

Previous contact with a different healthcare system can shape patients’ expectations, leading to frustration if they don’t receive the anticipated level of care. For example, a male respondent in his late 20s shared his experience contacting German healthcare:

After being accustomed to the Ukrainian healthcare system, where preventive treatment is common, I know that I need regular monitoring of my stomach, including annual blood tests and ultrasounds. I faced difficulties with this because here they said that I can’t have an ultrasound, only take some pills, but they didn’t explain why.

This experience underscores the challenges of adapting to a new healthcare system and highlights the participant’s unmet need for more information.

The reported differences in healthcare created significant barriers for some participants. A female participant in her late 40s summarized this issue as a clashing of expectations:

[…] Our people [Ukrainian] don’t always know what to expect when they visit a doctor here, and neither does a doctor know what a person might expect from the appointment, so this is probably the biggest problem. Sometimes, when a patient comes with different expectations from their home country and these expectations are not met, I think this is the biggest challenge for the patient and the doctor as well.

### Barriers to accessing the health care system and using health care services

The language barrier was one of the most significant obstacles in accessing and interacting with the German healthcare system mentioned among the study participants. Some participants cited this as the reason why they only visited Russian-speaking physicians in Germany. A female refugee in her late 40s explained that she relies on her son to communicate with German-speaking practitioners:

The language barrier is definitely a problem; that’s why I mostly visit Russian-speaking doctors or take my son as a translator with me.

Some participants who assisted elderly refugees or their refugee parents with health-related issues and acted as interpreters during medical appointments emphasized the importance of language proficiency and familiarity with the healthcare system for accessing care. For example, a young Ukrainian student who accompanied an elderly refugee with cancer to physician appointments shared her perspective:

Honestly, she wouldn’t be able to access medical services on her own. GP who speaks Russian, yes, but everything other - no. Even with such mundane things as calling a practice and booking an appointment, even with Google Translate, it is challenging […] without language skills, there is no way. You need to have resources, not only language but also certain experience: who do I need to contact, how is it organised here? Apart from language, there is a need for certain familiarity with the system, not only medical but also social system in Germany.

Many participants faced structural barriers realted to the availability of health services, such as long waiting times, lack of appointments, short working hours of outpatient practices, a shortage of specialized doctors in rural areas, and limitations of state insurance. Several respondents reported that they couldn’t get an appointment with a doctor for several months or at all. Some participants described healthcare as theoretically accessible “on paper” through insurance but difficult to access in practice due to these barriers.

Several refugee respondents reported significant barriers in accessing healthcare due to issues with social services and difficulties obtaining health insurance. A refugee participant diagnosed with cancer shared her experience of contacting social services and obtaining health insurance in the early days of the war. She had to apply for social benefits multiple times before being approved, and the process required her to wait in long lines for hours, sometimes even an entire day:

Just to get registered, it took me eight trips there. I was in decent condition; I was a year out of treatment at that time. I had the strength and resources to do it, and those people who couldn’t do it didn’t manage [to do it] in the end […] So, these first two months were missed for people who needed treatment; it just was discontinued, and they didn’t get it, that’s a fact.

This participant, a social activist who volunteers to assist other refugees with cancer diagnoses, explained that these obstacles led to disruptions in therapy for many cancer patients. Another female refugee participant described her struggle with accessing healthcare due to issues with social services:

I spent my treatment voucher on this visit but did not receive any help […] they took this voucher at the reception desk, so I can’t go to another doctor. The next time, I didn’t have the strength to stand outside for six hours in line for this piece of paper, and I can’t bear to think about it now; I want to cry because it was incredibly torturous, especially for a sick person.

The participant referred to the issuance of treatment vouchers by the social authorities in Germany, a process established during the refugee crisis of 2015/16 that was applied to Ukrainian refugees during first months after activation of temporary protection Directive.

### Previous experience with the German healthcare system

Participants shared various issues and personal stories regarding their medical history when asked about their experiences with the German healthcare system. In some cases, the patients subjectively experienced their acute health problem as more threatening and graver in comparison to the opinion of medical professionals. They expected comprehensive treatment and a more in-depth approach to their issues, leading to frustration upon not receiving the desired care. In a few instances, respondents felt that they had been prescribed the wrong therapy, and doctors did not perform diagnostics before the prescription. A female respondent in her early 20s described her experience:

I went to my GP, whom I eventually changed. I visited him regularly for a month, and each week, he prescribed me new antibiotics without doing any tests. So, for a whole month, I was on antibiotics without any betterment […] They haven’t had any interest in providing me with competent advice and told me to drink more tea.

In other instances, the patients revealed they did not receive any treatment and described physicians referring them to another specialist without trying to help. Some participants disclosed having a negative experience due to the rudeness of medical practitioners. A female participant in her middle 30s shared:

Yes, well, I had a miscarriage. The doctor traumatized me for a long time; she was very rude. She knew that I sought a second opinion from another doctor to check if there was a problem. When I returned to her, she was very rude to me […] it was very unpleasant.

Many participants heard at least one negative account of healthcare services in Germany from their friends or family. These respondents confirmed that such stories negatively influenced their expectations and opinions. Several participants mentioned that they put off or avoided doctor appointments because of negative expectations due to their friends’ poor experience with the healthcare system. A middle-aged female participant disclosed that such negative accounts led her to avoid seeking medical assistance in Germany:

Before I had any direct experience with the medical system in Germany, I heard that you couldn’t get to a doctor, so I put my problems on the back burner, thinking that as long as this is not an emergency, I don’t have to deal with it.

This experience underscores the complexity of building trust in a new healthcare system, highlighting that this process extends beyond individual experiences and is significantly shaped by collective perceptions and shared narratives.

### Suggested improvements to the German healthcare system

During the interviews, most participants enthusiastically contributed their ideas and recommendations to improve the German healthcare system.

Many participants highlighted the importance of interpersonal communication and mentioned they would like to see a more attentive and patient-centric approach. Some respondents noted they would like more information about their illness and treatment from their physician, underscoring the significance of shared decision-making. A female respondent in her mid-30s conveyed that she would like physicians to take more time to listen and explain:

Well, I look for a personalized approach when the doctor is interested in your story, can offer you several treatment options or can explain. Here, you are often just given a piece of paper with a prescription and do not understand why you were prescribed these medicines.

The participant criticized that she often felt she was being processed on a conveyor belt without being heard due to a lack of time and many patients. This illustrates how structural barriers might make patients feel ignored or give an impression of superficial treatment.

Many respondents underscored the need to improve its accessibility and attributed the existing lack of access to the aforementioned structural barriers. Many respondents saw further digitalization of healthcare as a possible solution. Several participants acknowledged the general shortage of doctors and other medical professionals as a potential reason for current problems in German healthcare and the need to solve this problem in the long term. One respondent suggested the integration of Ukrainian physicians into the healthcare system as a possible solution.

Another important topic mentioned was including psychological help while treating refugees, while a few respondents noted they would appreciate more tolerance and understanding towards Ukrainian refugees from medical personnel. A young female refugee who works as an interpreter in a refugee center shared her opinion on this topic:

I think the only thing we need is more tolerance for Ukrainians who know nothing about this life. This means tolerance from nurses, service staff, and doctors. Understanding that it is necessary to explain and that the process of understanding takes some time.

## Discussion

This study aimed to identify the main challenges and barriers experienced by Ukrainian refugees and migrants in accessing and utilizing healthcare services in Germany.

We found that one of the main barriers to access health services in Germany was the language barrier. These findings are consistent with previous research, as the linguistic barrier is one of the most common obstacles quoted in studies of migrants’ access to health services in European countries [5, 6, 11, 17].

Another prevalent barrier was the lack of understanding of the German healthcare system and the absence of sufficient information on the organization of the healthcare system. Though many participants found healthcare services in Germany accessible as covered by insurance, the lack of accessible information and understanding of the system posed a problem in accessing medical services. Similar findings were shown in the studies with Ukrainian refugees in Romania [10, 11].

It has been demonstrated that language and information barriers can cause incorrect treatment, inadequate care, and underuse of outpatient services, particularly for patients in vulnerable situations. These recognized challenges create hardships for healthcare providers and patients, resulting in unfulfilled healthcare needs [5, 28].

Following previous research on Ukrainian refugees [10], our study underscored the difficulties of adjusting to a different healthcare system. The Ukrainian system was the primary reference point for participants, and differences in referral processes, appointment scheduling, and consultation duration presented unique challenges. Previous research shows that the healthcare system’s structural aspects, such as variations in referral processes, can act as significant barriers, especially for individuals who have interacted with different healthcare systems [6]. Similar to other studies, perceived structural differences might prompt migrant and refugee participants to use transnational health services and seek help from healthcare providers in their home country [29–31].

Bureaucratic hurdles within the German healthcare system were a recurrent concern among participants. Several refugees, particularly those with severe conditions, faced a unique barrier in accessing healthcare due to difficulties in obtaining health insurance, possibly leading to delayed treatment and, consequently, poorer health outcomes. Similar structural hurdles were also reported within the Ukrainian refugee group in Romania [11]. This challenge may be related to the initial issue of treatment vouchers by the social authorities in Germany. Previous research has confirmed that this process creates additional barriers in accessing medical care [32]. As of 1 June 2022 Ukrainian refugees were granted unrestricted access to the German healthcare system through integration into the Social Code Book (Sozialgesetzbuch). This included access to electronic health cards, which replaced the problematic voucher system, eliminating additional barriers in accessing healthcare [33].

Another barrier to accessing healthcare services and patient satisfaction was a difference in treatment approaches and clashing perspectives towards healthcare. Similar results have been shown in studies with eastern European migrants in Scotland [29].

As Kleinman [34] observes, it is possible that healthcare providers do not always factor in their patient’s beliefs, past experiences, and values while giving advice or information. As a result, they might fail to understand that unconventional health-seeking behaviours, which could seem unreasonable or inconsistent, could stem from migrants’ previous experience with a different healthcare model or system shaped by different ideologies, values, and priorities. This mutual lack of comprehension can lead to mistrust between patients and healthcare providers. Consequently, migrants or refugees might feel ignored or unfairly treated.

As demonstrated in studies from other countries, there appears to be a complex interaction of factors at play when it comes to the reported lack of trust that Ukrainian refugees have in the German healthcare system. Cultural disparities lead to miscommunication and discontent, reflected in different patient expectations and healthcare procedures [29]. Effective interactions between immigrants and healthcare providers are hampered by communication challenges primarily caused by language barriers [35]. It has been shown that miscommunication can lead to multiple adverse consequences: disruption in ongoing care, risks to patient safety, dissatisfaction among patients [6], and an inefficient allocation of important resources, which may lead to economic losses [36]. Furthermore, a lack of consultation time may make it difficult for patients and medical professionals to communicate and understand one another. This might lead to distrust as the patient may feel not taken seriously [6]. A comprehensive strategy is required to guarantee a healthcare setting that is both culturally sensitive and helpful in building trust among Ukrainian refugees in Germany. Interventions should include language support services, cultural competency training for healthcare professionals, increases in the healthcare system’s efficiency to ensure optimal use of resources, patient-centred care, and communication initiatives, including providing better information about the healthcare system, to address these problems and restore trust [10]. Difficulties navigating the system and the alleged deficiency of individualized treatment also point to areas where healthcare service delivery could be improved. The intricate process of trust-building extends beyond personal experiences to encompass perceptions and narratives shared among individuals. Given this complexity, future research needs to delve deeper into and expand upon trust in healthcare professionals among the Ukrainian refugee population.

This study may offer relevant insights for other countries managing a significant influx of Ukrainian refugees. However, as inter-agency data exploration indicates [37], the implementation of the Directive on temporary protection varies by country as do health systems. Therefore, the findings may not apply to all Ukrainian refugees across Europe. It is also important to note that Ukrainian refugees have a unique legal status due to the temporary protection Directive. In Germany this grants them broader access to healthcare and social services compared to refugees/asylum seekers from other countries [4, 38], making the legal context for policy and program solutions unique to this group.

### Study limitations

The primary limitation of the study was the representativity of the sample. Recruitment through social media platforms may have led to the underrepresentation of the older population in our sample. Taking into consideration this cohort might be more prone to suffer chronic conditions and have different experiences with the healthcare system, further research is needed to assess barriers this group might face. Furthermore, as martial law prohibits adult men from leaving Ukraine, the male sex is underrepresented in our study population.

Given the relatively large sample of 30 participants for a qualitative study, we feel that we achieved sufficient data saturation. However, we acknowledge that other migrants and refugees may have different experiences with the German healthcare system.

## Conclusion

This study underscores the importance of addressing cultural and structural barriers to improve healthcare accessibility and utilization for Ukrainian refugees and migrants in Germany. The findings also highlight the need for increased tolerance and understanding of the challenges faced by these individuals. It is recommended that improvements be made in communication, including interpretation services and the provision of comprehensive information about the healthcare system. Additionally, efforts to simplify bureaucratic procedures related to obtaining health insurance and facilitate more unrestricted access to primary care could prove beneficial.

This study contributes to a deeper understanding of the obstacles faced by Ukrainian refugees and migrants. These findings offer valuable insights for policymakers and healthcare providers to develop effective strategies for improving healthcare accessibility and utilization for migrant and refugee populations. However, additional research is necessary to explore these challenges and potential solutions further, particularly as the migrant and refugee populations evolve.

## Supplementary Information

Additional file 1.  Interview Guide.  The detailed interview guide used while performing the interviews.

## Data Availability

Detailed interview guide is available in the supplementary information files.The datasets generated and analysed during the current study are available from the corresponding author upon reasonable request.
