# Supplementary material for: Barriers of Ukrainian refugees and migrants in accessing German healthcare
Source: BMC Health Serv Res. 2024 Sep 24;24:1112. doi: 10.1186/s12913-024-11592-x (PMC11423494; doi:10.1186/s12913-024-11592-x)
Supplement: Supplementary file 1 — Additional file 1. Interview Guide. The detailed interview guide used while performing the interviews. [file 12913_2024_11592_MOESM1_ESM.pdf]

Interview Guide

Framework conditions

- Semi-structured interview
- Open questions as far as possible
- The objective of the interview guide is to cover all aspects relevant to the research topic through various questions.
- The order of the topics should be maintained as far as possible.
- The order of the questions depends on the course of the interview.

Research question:

Barriers of Ukrainian refugees and migrants in accessing German healthcare.

Beginning of the interview

- Greeting and introduction of the interviewer
- The interviewer is a scientist and has the same cultural and religious background as the participant.
- Thank the participant for their willingness to take part in the interview.
- A brief explanation of the topic of the interview
- Description of the procedure and the approximate duration of the interview
- Information about data protection regulations and informed consent for the interview

Introductory question

Should be asked as openly as possible and create a pleasant atmosphere, serves as an introduction to the interview.

Main part

- Contains key questions on the research topic.
- Questions should be as open as possible.
- Follow-up questions can be noted in advance or arise from the course of the interview.
- The guideline is structured based on the questions relevant to the research topic to keep these in focus.

Topics of the main section

1. Demographic data
   - How long have you been in Germany?
   - How old are you?
   - Which gender do you feel you belong to?
   - What is your highest level of education?
   - Which languages do you speak and at what level?
   - What profession did you learn/what profession do you practise?
   - Do you live alone/together with your family? Do you have children?
   - Are you religious? Which religion do you belong to? How big a role does religion play in your everyday life?
   - Do you have any pre-existing conditions and/or chronic illnesses?

2. Obtaining information on health topics and services, knowledge about the functioning of the health care system in Germany
   - From whom/where do you receive information about the health, illness, and treatment?
   - Who do you trust the most when it comes to your health?
   - There are different situations that can cause health problems. Who do you turn to with different problems (doctor, emergency department)?

3. Barriers to accessing the health care system and using health care services.
   - What obstacles do you face in obtaining medical services in Germany?
   - How would you rate the availability/accessibility of medical services for you?
   - Do you think cultural differences would be a problem while obtaining medical services?

4. Past experiences with the health care system in relation to their health/illness and in general
   - What problems do you have regarding your physical and mental health?
   - Which part of the health care system (physician, hospital, ...) did you visit and what kind of therapy did you receive?
   - Do you feel that you have received full treatment?
   - Has your doctor informed you sufficiently about your illness, and did they include you in the therapy decision?

5. Expectations of the healthcare providers and healthcare system
   - What expectations do you have towards healthcare providers?
   - How satisfied have you been in situations where you have used the healthcare system?
   - Which part of the health care system do you/your family trust most?
   - If you have had a negative experience with the health system, what happened/what was the problem?
   - What experiences with the health system have friends/relatives had? Does this experience influence your expectations (negatively or positively)?
   - What improvements would you like to see in the healthcare system and its accessibility?

<u>End of the interview</u>

- Thank the participant for taking part in the interview.
- Explain that the study can contribute to the improvement of health care for refugees in Germany.
- Farewell
